# Three-Dimensional Finite Element Method Simulation of Perforated Graphene Nano-Electro-Mechanical (NEM) Switches

**DOI:** 10.3390/mi8080236

**Published:** 2017-07-31

**Authors:** Mohd Amir Zulkefli, Mohd Ambri Mohamed, Kim S. Siow, Burhanuddin Yeop Majlis, Jothiramalingam Kulothungan, Manoharan Muruganathan, Hiroshi Mizuta

**Affiliations:** 1 Institute of Microengineering and Nanoelectronics, Universiti Kebangsaan Malaysia, 43600 UKM-Bangi, Selangor, Malaysia; amir.zulkefli90@siswa.ukm.edu.my (M.A.Z.); kimsiow@ukm.edu.my (K.S.S.); burhan@ukm.edu.my (B.Y.M.); 2 School of Materials Science, Japan Advanced Institute of Science and Technology, Nomi, Ishikawa 923-1292, Japan; jothi@jaist.ac.jp (J.K.); mano@jaist.ac.jp (M.M.); mizuta@jaist.ac.jp (H.M.)

**Keywords:** nanoelectromechanical (NEM) switch, graphene, intact beam structure, perforated beam structure, finite element simulation (FEM), pull-in voltage characteristic, von Mises stress

## Abstract

The miniaturization trend leads to the development of a graphene based nanoelectromechanical (NEM) switch to fulfill the high demand in low power device applications. In this article, we highlight the finite element (FEM) simulation of the graphene-based NEM switches of fixed-fixed ends design with beam structures which are perforated and intact. Pull-in and pull-out characteristics are analyzed by using the FEM approach provided by IntelliSuite software, version 8.8.5.1. The FEM results are consistent with the published experimental data. This analysis shows the possibility of achieving a low pull-in voltage that is below 2 V for a ratio below 15:0.03:0.7 value for the graphene beam length, thickness, and air gap thickness, respectively. The introduction of perforation in the graphene beam-based NEM switch further achieved the pull-in voltage as low as 1.5 V for a 250 nm hole length, 100 nm distance between each hole, and 12-number of hole column. Then, a von Mises stress analysis is conducted to investigate the mechanical stability of the intact and perforated graphene-based NEM switch. This analysis shows that a longer and thinner graphene beam reduced the von Mises stress. The introduction of perforation concept further reduced the von Mises stress at the graphene beam end and the beam center by approximately ~20–35% and ~10–20%, respectively. These theoretical results, performed by FEM simulation, are expected to expedite improvements in the working parameter and dimension for low voltage and better mechanical stability operation of graphene-based NEM switch device fabrication.

## 1. Introduction

The success of the semiconductor industry depends on reliable performance and scalable manufacturing processes. As the size of semiconductor devices miniaturize to a few tens of nanometers and the demand for more applications keeps increasing, another strategy will be needed. A nanoelectromechanical (NEM) switch is one of the promising devices to solve the problems of high power consumption in complementary metal-oxide semiconductor (CMOS) circuits [1,2]. The NEM switch is built into logic circuits, relays, data storage, and high frequency communication because of its high ON-OFF current ratio and low leakage current [3,4,5]. However, the conventional NEM switch still underperforms compared to the conventional semiconductor switch because of low reliability and a high actuation pull-in voltage [6]. There are two common ways to reduce the mentioned problems such as the introduction of new materials and proper geometrical design [7,8].

Graphene is one of the suggested 2D materials for high-performance NEM switch application because of its superior properties namely high electron mobility excess of 200,000 cm^2^/V·s, high Young’s modulus of 1 TPa, superior current density capacity of 10^8^ A/cm^2^, the ultra-thin thickness of 0.335 nm and low resistivity of 1 µΩ·cm [9,10,11,12]. For these reasons, graphene-based NEM switch can provide better reliability and lower actuation pull-in voltage than a conventional switch [13]. An optimized geometrical design of the NEM switch can also achieve the same objectives of better reliability and low actuation pull-in voltage. In the past few years, the geometrical design of the graphene switch has changed to an actuated bottom and top electrode to improve its mechanical stability and actuation pull-in voltage [14,15,16]. Others introduced the concept of perforation in the beam structure of radio frequency microelectromechanical system (RF MEMS) switch to increase the beam flexibility, lower the actuation pull-in voltage, and enhance the switching speed [17,18]. There is also previous work which achieved different shapes of perforation in graphene sheets through experimental work [19]. However, to the best of our knowledge, the similar perforation concept was not introduced in the graphene beam based NEM switch application to reduce the actuation pull-in voltage.

In this work, we emphasize on the actuation pull-in voltage and von Mises stress reduction for a graphene-based NEM switch by using 3D finite element (FEM) modeling and simulation of a multilayer graphene beam with and without the perforated structure of different geometrical dimensions. We concentrate on optimizing the design of the graphene-based NEM switch in-line with the experimental work [20]. In addition, the characteristics of pull-in and pull-out voltage from the intact graphene simulation results are consistent with the experimental data. Then, we used these models to study the intact beam and perforated beam scaling on switching characteristics. We evaluated the mechanical stability of the NEM switch by von Mises stress analysis after validating the FEM simulation results with the published experimental data for the pull-in characteristic of the fixed-fixed ends graphene-based NEM switch. Moreover, the influence of the applied electric field on the intact and perforated graphene-based NEM switch were also analyzed.

## 2. Methodology

### 2.1. Description of Device Geometry and Operation Principles

**Figure 1:**
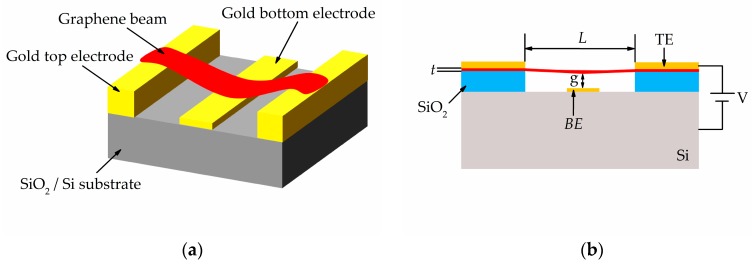
Graphene-based nanoelectromechanical (NEM) switch with bottom actuation electrode: (**a**) schematic diagram; (**b**) side view.

**Figure 2:**
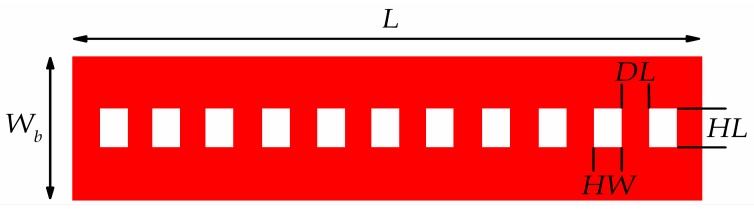
Top view of graphene beam structure.

This section concisely explains the geometry of NEM switch and its operation principles. Firstly, the switching operation of multilayer graphene-based NEM switch was studied based on the previous experiment work [20]. The multilayer graphene or also known as nanocrystalline graphene (NCG) was synthesized by using plasma-enhanced chemical vapor deposition (PECVD) [21] and this deposited NCG have both sp^3^- and sp^2^-hybridized carbon atoms. Besides, this NCG film is polycrystalline in nature [20]. We defined this multilayer graphene as an isotropic material in this FEM simulation of graphene-based NEM switch as it is a polycrystalline material. We used the device structure and dimensions from previous work to validate our proposed model [20]. Based on this experimental work, we used 860 GPa as the Young’s modulus for graphene in all the FEM simulations which are comparable to the values of 500 GPa measured for the multilayer graphene between 2 to 8 nm of thickness [22]. Afterwards, we studied the design parameters of the NEM switches, such as, graphene beam length *L*, thickness *t*, and air gap thickness *g* because of their influence on the mechanical and electrical properties of the switch. The length and thickness of the graphene beam were varied from 0.8 µm to 1.5 µm and 3.0 nm to 9.0 nm, respectively. The air gap thickness was varied from 50 nm to 130 nm. It is worth to mention that the surface adhesion effect between graphene beam and bottom electrode contact is neglected as the influence of surface effect such as van der Waals force becomes insignificant as the device size increases. It is known to be dominant when the air gap thickness, *g* < 20 nm [23]. The optimized design from this initial model was then re-analyzed with a perforated graphene beam. In the perforated graphene beam, we varied the hole length *HL*, hole width *HW*, distance between two holes *DL* and number of hole column *CN*. This perforation concept is expected to reduce the biaxial residual stress and the graphene beam stiffness, thus contributing to the reduction of graphene beam buckling-effect to increase the switch lifetime and reduced actuation pull-in voltage [24]. The schematic configuration and side view of the graphene-based NEM switch is illustrated in Figure 1a,b respectively. While its device dimensions are detailed in Table 1, and the top view of the perforated graphene-based NEM switch is shown in Figure 2.

**Figure 3:**
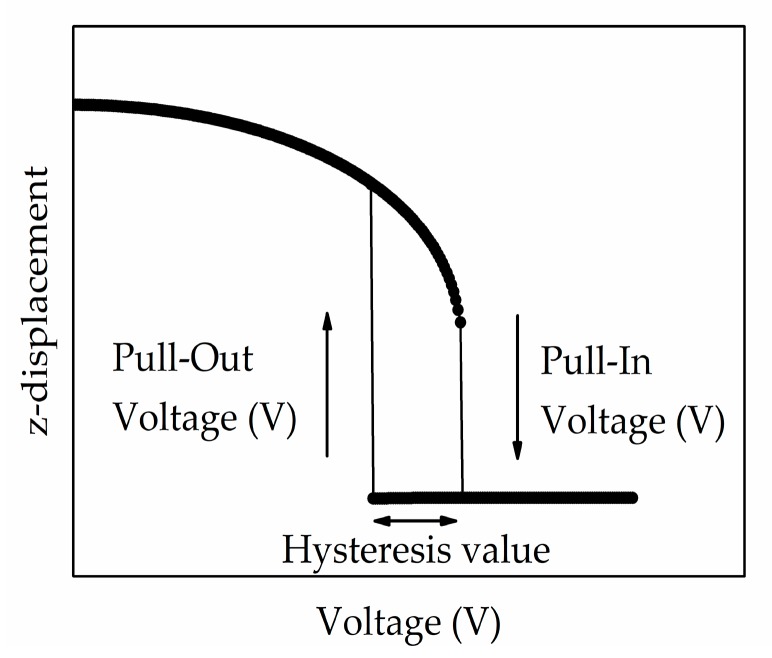
Typical actuation voltages characteristics for pull-in, pull-out voltages, and hysteresis of the graphene-based NEM switch.

The operation of graphene-based NEM switch depends on the distance and corresponding electrostatic force between the bottom electrode and suspended graphene beam. The voltage between the gold bottom electrode and the graphene beam is increased until it reaches a critical point known as pull-in voltage. At this stage, the electrostatic force overwhelms the elastic restoring force of the deformed graphene beam to accelerate towards the bottom actuation electrode, resulting in closing the device which leads to a sharp rise in the current flow through the device [25]. As the voltage is decreased below the critical point, now known as pull-out voltage, the elastic restoring force in the deformed graphene beam overwhelms the electrostatic force, which restoring the graphene beam to its original state and thus, creating an ‘off-state’ mode for the switch. A common actuation pull-in, pull-out voltage and hysteresis characteristics are illustrated in Figure 3.

### 2.2. Thermo-Electromechanical (TEM) Analysis Model (FEM-IntelliSuite)

In our simulation work, the FEM-based CAD tool IntelliSuite (8.8.5.1, IntelliSense, Lynnfield, MA, USA) [26] under the thermo electromechanical (TEM) analysis model was used to obtain the actuation pull-in voltage and the graphene beam deflection. The TEM model is the integration of two different modules, namely Thermo-Electrical module and Electro-Mechanical module. The Electro-Mechanical module was only used in this analysis as we are not discussing problems associated with heat which is related to Thermo-Electrical module. The model structure of graphene-based NEM switch is mechanically meshed with adaptive mesh to refine the deflection of the graphene beam. In IntelliSuite, all the individual elements are treated as free elements (free to move in all the spatial coordinates, *x*, *y*, and *z*). Therefore, we need to set our model boundary conditions to get the accurate results. There are two different boundary conditions used in our IntelliSuite software analysis namely the fixed boundary condition and the contact pair boundary condition. The fixed boundary condition was set at the both ends of graphene beam, and the bottom surface of the model geometry. While, the contact pair boundary condition was set at the top of the bottom electrode surface and the bottom of the graphene beam surface. In general, we used the Face pair type of the contact pair to find the pull-in voltage, where the suspended graphene beam deflection will be stop at a specific spatial coordinate value. For the electrostatic force domain, the following equation is solved for the electromechanical problem in TEM module by IntelliSuite software for each discretized element.

ϕ (x) = *∫_surface_G*(*x*, *x*′) *σ*(*x*′) *da*′, (1)

where ϕ is the surface potential, *σ* is the surface charge density, *G*(*x*, *x*′) is the green function, and *da*′ is incremental in the surface area. The following equations were used by IntelliSuite software to solve the problem for the mechanical domain.

(2) $$\left\{ \begin{array}{l} K_{qq}q=F_{ele}(V)\\ K_{uu}(u)=F_{boundary}(V)\\ K_{vv}(u,V)V=V_{boundary} \end{array}\right.,$$

The first equation is the purely mechanical system which considering the electrostatic forces, *F_ele_*. Parameter, *q*, in the first equation is the vector containing the displacements of the mechanical structure. This equation also known as the equilibrium equation of the mesh. While, the vector *u* in the second and third equations are the displacement of the electrostatic mesh nodes. The third equation is the electrostatic system which depending on the vector, *u*, displacement. Finally, the electro-mechanical coupling appears through this deformed mesh.

### 2.3. Empirical Equation of Actuation Pull-In Voltage

The actuation pull-in voltage empirical equation for a fixed-fixed end graphene beam NEM switch is given by [27,28]:

(3) $$V_{pi}=\sqrt{\frac{8Kg^{3}}{27\varepsilon_{0}W_{b}W_{c}}},\;K=\sqrt{\frac{2Kgd^{2}}{\varepsilon_{r}^{2}\varepsilon_{0}W_{b}W_{c}},}$$

where *g* is the air gap between the graphene beam and bottom actuation electrode, *K* is the beam spring constant, *ɛ*_0_ is the vacuum permittivity of 8.853 × 10^12^ F·m^−1^, *L* and *W_b_* are the graphene beam length and width, respectively. Besides, *W_c_* is the contact width of the bottom electrode, *E* is the Young’s modulus of graphene, and *t* is the graphene thickness which is thoroughly related to the number of graphene layer. The derivation of this pull-in voltage equation can be found in [27,28]. The Equation (3) here is for a fixed-fixed end graphene beam under the pure bending by a uniformly distributed load.

The actuation pull-in voltage equation of perforated graphene beam NEM switch is also specified by Equation (3) but with modification of the beam spring constant because of the changes in its Young’s modulus which corresponding to the beam volume and mass. The typical Young’s modulus, stress and strain equations of a beam structure are given by [29]:

(4) $$E=\frac{\sigma }{\varepsilon },\;\sigma =\frac{F}{A},\;\varepsilon =\frac{\Delta l}{l_{o}},$$

where stress, *σ*, is defined as the applied force per unit original under deformed cross-sectional area of the perforated graphene. While strain, *ɛ*, is the engineering strain defined as the change of graphene length during ‘on-state’ mode per unit of the initial graphene length during ‘off-state’ mode. The applied force upon an object is based on second law of motion given by [30]:

*F* = *ma*, *m* = *ρV*, (5)

where *ρ* is density of the beam defined as beam mass per unit volume of the beam. While *a* is the acceleration of the beam. We can assume that the perforated graphene beam volume is defined as the effective volume of graphene beam after deducting the volume of holes.

## 3. FEM Simulation Results and Discussion

### 3.1. Analysis of Actuation Pull-In Voltage of Intact Graphene-Based NEM Switch

**Figure 4:**
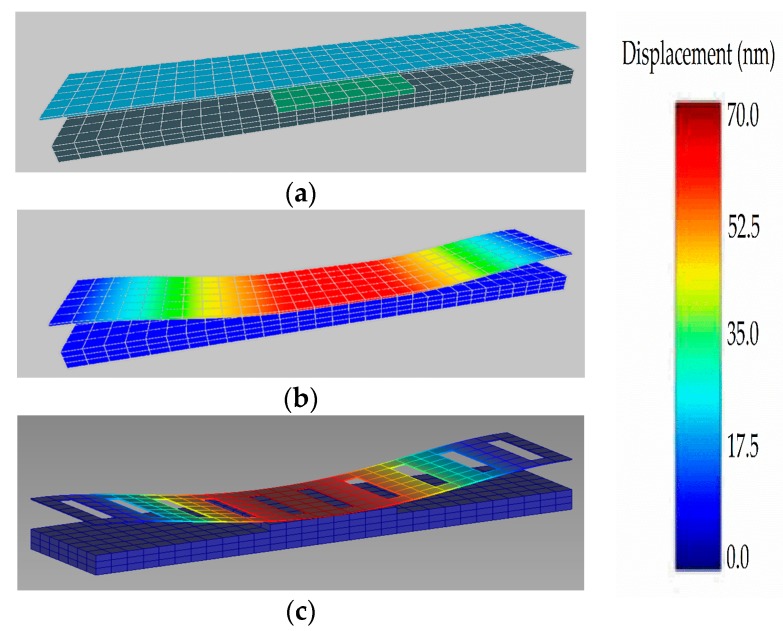
Graphene-based NEM switch with bottom actuation electrode during: (**a**) ‘off-state’ mode; (**b**) ‘on-state’ mode; (**c**) ‘on-state’ mode for the perforated graphene beam.

**Figure 5:**
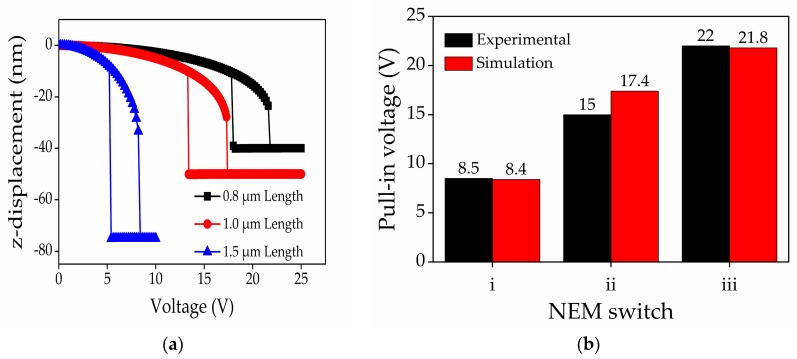
(**a**) Simulation data of actuation pull-in voltage characteristics for fixed-fixed ends graphene-based NEM switch; (**b**) comparison of pull-in voltage between experimental and simulation data for model validation process.

First, the device dimensions mentioned in Table 1 were used in order to be consistent with the previous experimental device structure [20]. Then, we compared the actuation pull-in voltage characteristics obtained for the different dimension of a fixed-fixed ends graphene-based NEM switch with the previous experimental results to validate our model. In the meantime, the original geometry of an intact graphene-based NEM switch used in this FEM simulation work was illustrated in Figure 4a. The applied voltage between the graphene beam and the gold bottom electrode was accelerated until the pull-in mode was confirmed, and then, it decreased back to 0 V. Figure 4b,c show the geometrical configuration of the intact and perforated graphene-based NEM switch under the ‘on-state’ mode, respectively. The color bar illustrated the graphene beam displacement with respect to the original position. The actuation pull-in voltages for the respective dimensions in the NEM switch i, ii, iii shown in Figure 5 and Table 1 were approximately 8.4 V, 17.4 V, and 21.8 V, respectively. These results are consistent with the previous experimental data [20].

**Figure 6:**
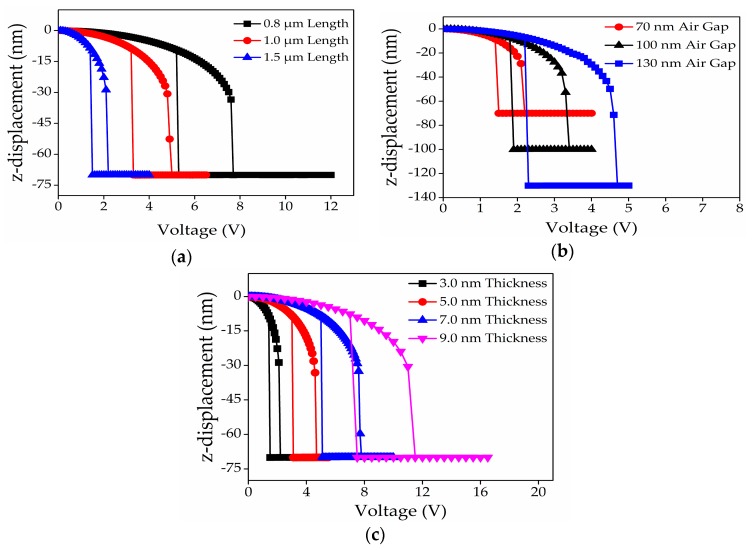
Actuation pull-in voltage characteristics of a fixed-fixed ends graphene-based NEM switch for variation of: (**a**) beam length; (**b**) air gap thickness; (**c**) beam thickness.

In addition, we conducted an FEM scaling analysis on these validated models, as tabulated in Table 2, to further clarify the influence of the graphene beam length, thickness, and air gap thickness on the hysteresis, pull-in and pull-out voltages. Figure 6a–c show the actuation pull-in voltage behaviors of the graphene-based NEM switch with the variation of the graphene beam length, thickness and air gap thickness, respectively. Figure 6 clearly shows that a small air gap on long and thin graphene beam reduce the actuation pull-in voltage. Long and thin graphene beam reduced the beam stiffness and increased its flexibility, thus reducing its actuation pull-in voltage. Reduced air gap resulted in a decrease of pull-in voltage because of the increased electrostatic force between the graphene and bottom electrode.

**Figure 7:**
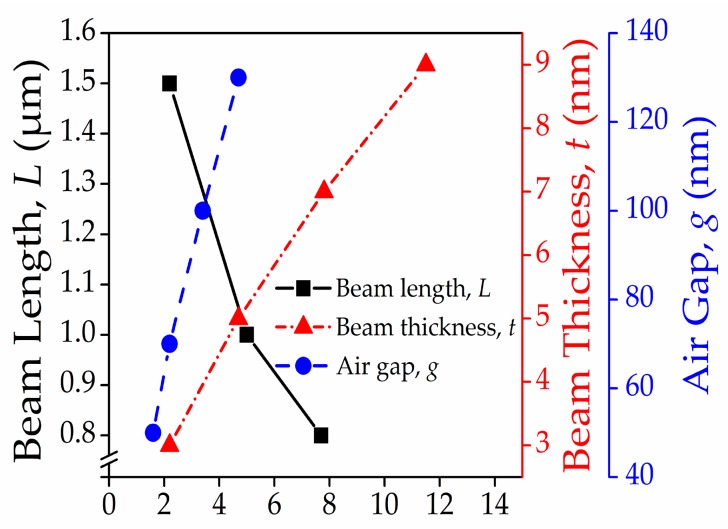
Trends of actuation pull-in voltage against beam length, beam thickness, and air gap thickness of the graphene-based NEM switch device.

**Figure 8:**
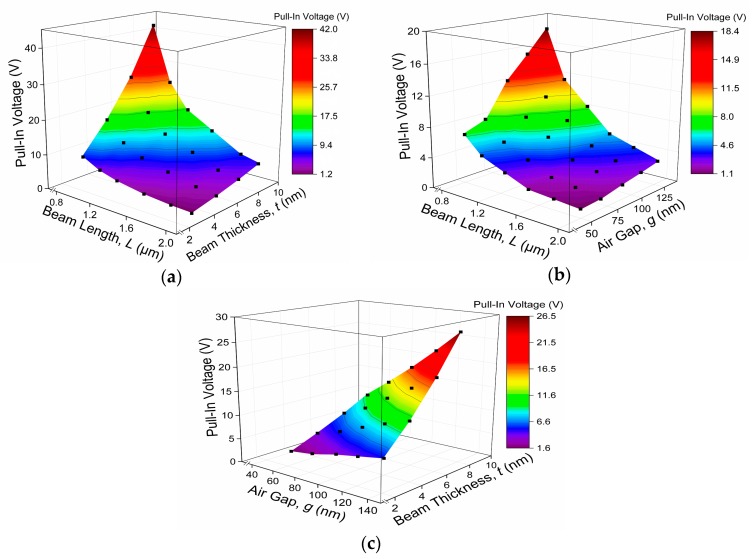
Trends of actuation pull-in voltage dependence of graphene-based NEM switch on: (**a**) beam length and beam thickness; (**b**) beam length and air gap thickness; (**c**) air gap thickness and beam thickness.

The results are summarized in Table 2 and re-plotted in Figure 7. Figure 7 shows that the relationship between the actuation pull-in voltage and the beam length is a negative correlation. The other two beam dimensions which are beam thickness and air gap show the positive correlation with the actuation pull-in voltage. Besides single-factor analysis, we also consider the dependence of actuation pull-in voltage on dual-factors namely; (a) graphene beam length and graphene beam thickness; (b) graphene beam length and air gap; (c) air gap thickness and graphene beam thickness. According to the dual-factor analysis in Figure 8a–c comparable actuation pull-in voltage of less than 2 V, are achieved at a ratio below 15:0.03:0.7 value for the graphene beam length, thickness, and air gap thickness, respectively.

### 3.2. Analysis of Actuation Pull-In Voltage of the Perforated Graphene-Based NEM Switch

Here, we evaluate the effect of perforation in the graphene beam on the actuation pull-in voltage of the NEM switch. The devices dimensions such as hole length (*HL*), hole width (*HW*), distance between two holes (*DL*), and number of hole column (*CN*) are tabulated in Table 3. We adopted the graphene beam length, thickness, and air gap thickness based on NEM switch-iii (Table 2) from the previous intact graphene beam analysis because of its optimized actuation pull-in voltage.

**Figure 9:**
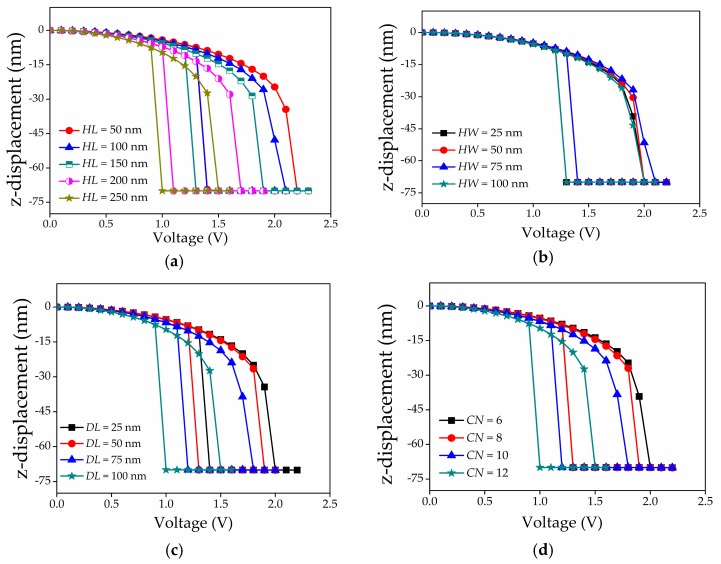
Actuation pull-in voltage characteristics of perforated graphene-based beam NEM switch with variation of: (**a**) hole length; (**b**) hole width; (**c**) distance between two holes; (**d**) number of hole column.

**Figure 10:**
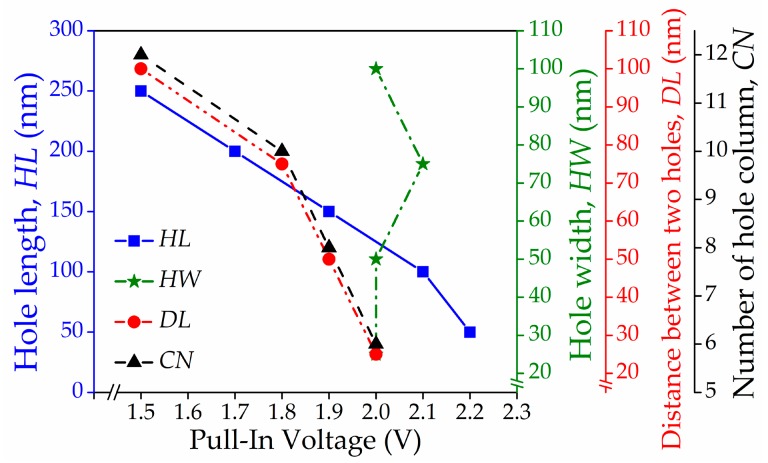
Trends of actuation pull-in voltage against hole length, hole width, distance between two holes, and number of hole column of graphene-based NEM switch device.

**Figure 11:**
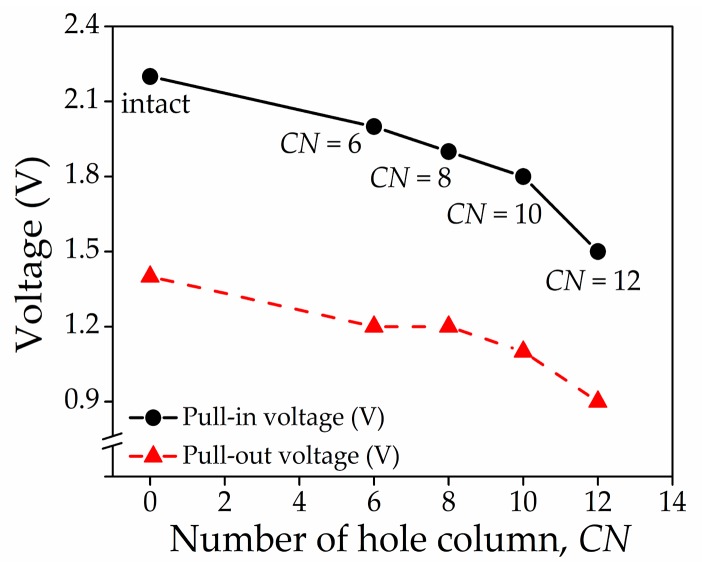
Comparison of actuation pull voltage characteristics between intact and perforated graphene-based NEM switch.

Figure 9a–d shows the actuation pull-in voltage characteristics for the perforated graphene beam with variations in *HL*, *HW*, *DL*, and *CN*, respectively. The results are tabulated in Table 3, and their trends can be observed in Figure 10. The trends show that *HL*, *DL*, and *CN* were negatively correlated to the actuation pull-in voltage. Then, the actuation pull-in voltage between intact and the perforated graphene beam NEM switch is compared in Figure 11. The results showed that the introduction of the perforated graphene beam structure reduced the actuation pull-in voltage by approximately ~9–32%. The perforation concept in the graphene beam structure reduced the beam mass and spring constant that lead to low beam stiffness. Simultaneously, the perforation eased the beam deflection and reduced the actuation pull-in voltage. The variation of *HL*, *DL*, and *CN* reduced the actuation pull-in voltage because of the reduction in beam mass and volume which changed the beam spring constant corresponding to Young’s modulus of the graphene beam. In addition, the pull-in voltage value with lower *DL* dimension can still be reduced by adding the holes in the graphene beam as more spaces becomes available with the decrease in *DL*, thus reducing the graphene beam stiffness.

However, the variation of *HW* did not affect the actuation pull-in voltage because the beam stress was only induced at the cross-section area of the beam thickness and *HL* without involving the *HW*. The hysteresis value is also reduced by approximately ~25% for the 12-column perforated graphene beam structure, 1.5 µm of graphene beam length, 3.0 nm of graphene beam thickness, and 70 nm of air gap thickness. This reduction was attributed to the reduction in effective area at the contacts interface between the perforated graphene beam and bottom actuation electrode. We also validated these models with the analytical expression by calculating the pull-in voltages using Equations (3) and (4) based on the perforated NEM switch dimensions in Table 3. The trends from the analytical models compared well with the FEM simulations results for the perforated graphene-based NEM switch.

### 3.3. Analysis of Von Mises Stress

One of the methods used to estimate the yield of ductile material such as metals is the von Mises stress analysis [31]. Failure theories for the yield of ductile materials are based on the shear or distortion. The maximum-distortion-energy theory equates the distortion energy for a general case of stress to the distortion energy when a simple tensile specimen yields. As the von Mises stress reaches the material yield strength, the maximum-distortion-energy theory forecasts the elastic failure of the material. The von Mises stress, *σ_vm_* is given by [32,33]:

(6) $$\sigma_{vm}=\sqrt{0.5\left[{\left(\sigma_{x}-\sigma_{y}\right)}^{2}+{\left(\sigma_{z}-\sigma_{y}\right)}^{2}+{\left(\sigma_{z}-\sigma_{x}\right)}^{2}\right]},$$

where *σ_z_*, *σ_y_*, and *σ_x_* are the stress in the *z*, *y*, and *x* Cartesian coordinate system, respectively. The mechanical stability of the graphene-based NEM switch can potentially be enhanced by optimizing the dimensions of the switch. The maximum von Mises stress employed across the graphene beam length was compared in order to demonstrate the mechanical stability of the graphene-based NEM switch quantitatively [34]. In addition, the profile analysis of von Mises stress is vital to realize the spatial variation generated on the graphene beam owing to the applied voltage. The system of a Cartesian coordinate is used to denote the numerical coordinates on the graphene beam. Besides, the profile of the stress was acquired after the pull-in mode was achieved, giving a 3-dimensional stress profile of the deformed graphene based on the constant variation of thickness and stress.

**Figure 12:**
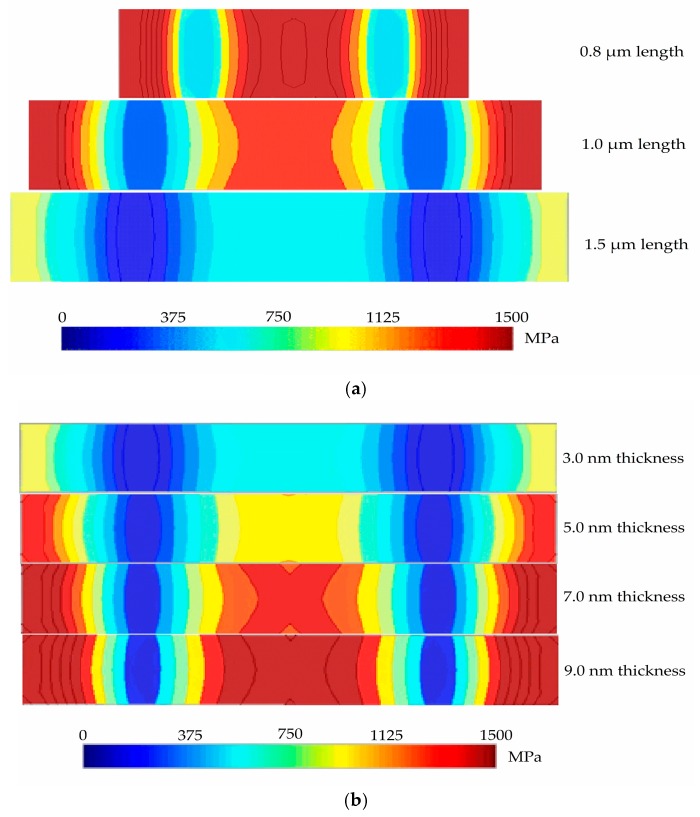
Von Mises stress contour plot from the top view (unit: MPa) with a variation of: (**a**) intact graphene beam length; (**b**) intact graphene beam thickness.

We carried out another FEM scaling analysis by using IntelliSuite software to justify the influence of *L* and *t* on the von Mises stress. Figure 12a,b illustrate the contour plot analysis from the top view of von Mises stress of the intact graphene-based NEM switches with variation in *L* and *t*, respectively. The maximum von Mises stress was achieved at the graphene beam ends when *t* and *L* was increased and decreased, respectively, due mainly to the increased electrostatic force between the gold bottom electrode and graphene beam. Based on Figure 12, the NEM switch with the thinner and longer graphene beam has the minimum probability of device failure.

**Figure 13:**
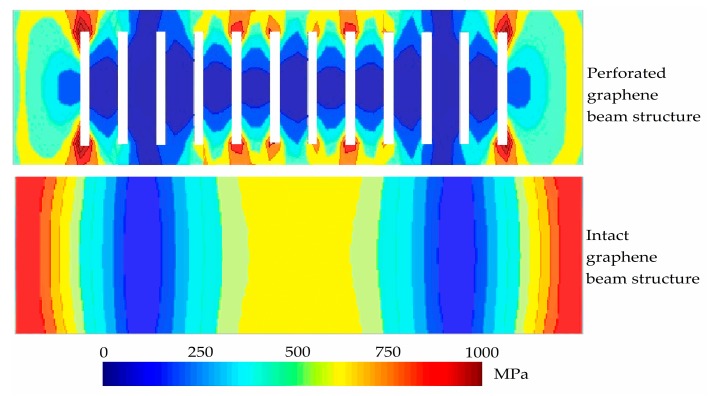
Comparison of von Mises stress contour plot from the top view (unit: MPa) between intact and perforated graphene-based NEM switch.

We further extend our FEM scaling analysis to elucidate the effect of the perforation concept in the graphene beam on the von Mises stress and compared it with the intact graphene beam structure analysis. The value of *L* and *t* were adopted from our previous optimized intact graphene beam analysis. Figure 13a,b illustrate the contour plot analysis from the top view of von Mises stress of the perforated and intact graphene-based NEM switches, respectively. The results from the contour plot confirm that the von Mises stress is highest nearer to both fixed ends of the intact graphene beam. For the case of perforated beam analysis, the von Mises stress is highest at the hole edges nearer to the beam ends and beam center because of the accumulation of electrical field distribution and the edge termination effect, where the electrical field strength is extremely concentrated at the hole edges and beam ends. Then, three-dimensional electrical field distributions in the graphene-based NEM switch were conducted in the pull-in mode in order to justify the previous von Mises stress results.

### 3.4. Analysis of Electrical Field Distribution

We used the exact model in COMSOL Multiphysics version 5.2 (COMSOL Inc., Burlington, MA, USA) [35] to investigate the influence of the electrical field distribution on the von Mises stress of the perforated and intact graphene-based NEM switch. The switch model used the electromechanics interface to solve the coupled equations for the structural deformation and the electric field. In the meantime, we modeled only half of the graphene beam because the geometry is symmetrical. The graphene beam was set with a Poisson’ ratio, *v*, of 0.17 and a Young’s modulus, *E*, of 860 GPa. The free-space environment was used in the model of the NEM switch. We used free tetrahedral triangular mesh elements to mesh the switch model to simplify computational complexity. The mesh density was varied adaptively to analyze the graphene beam structural displacement. In this analysis, the graphene beam and the gold electrode was positioned at the top and the bottom, respectively. The applied voltage was swept at the graphene beam and 0 V of fixed bias was applied to the gold bottom electrode to analyze the electrical field distribution at different voltages, while other boundaries are electrically insulated. Besides, the voltage potential (*V*) and the electrical field (*E*) in the vacuum condition can be acquired by solving the Poisson’s equation as given by [36]:

−∇·(ε·∇*V*) = 0, (7)

where the derivatives are taken with respect to the spatial coordinates. This numerical model represents the electrical potential and its derivatives on a mesh, which is moving with respect to the spatial frame. In addition, the required transformations are taken care of by the electromechanics interface, which consists the smoothing equations governing the mesh movement in the air domain.

**Figure 14:**
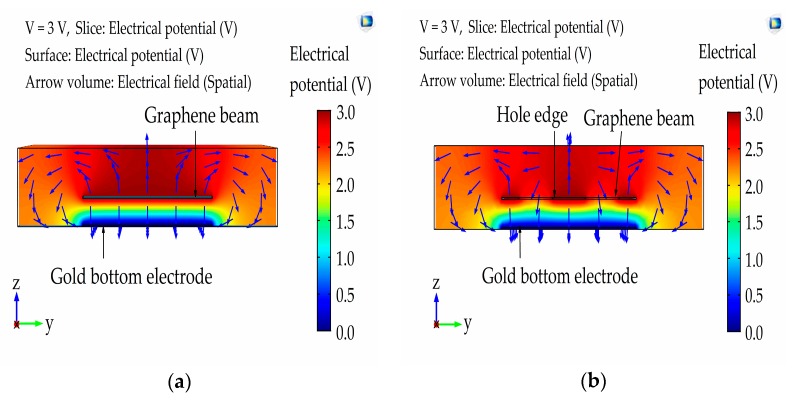
The 2-D electrical field distribution across the cross-sectional view of the graphene beam center. Arrows designate the electrical field direction: (**a**) intact graphene beam structure that used the dimensions of NEM switch-iii taken from Table 2; (**b**) perforated graphene beam structure that used the dimensions of NEM switch-E taken from Table 3.

Figure 14 shows the electrical field distribution during the pull-in state. The Figure is in a cross-sectional view across the graphene beam center with an applied voltage of 3 V. The arrows designated the electrical field lines, and are vertically distributed at the graphene beam center. In addition, the electrical field distribution is highly circulated horizontally from the graphene beam center to both of the graphene beam edges in the outward direction. The electrical field distribution is slowly altered to the horizontal direction along the beam edges and the hole edges, showing the reason for high von Mises stress at these locations.

**Figure 15:**
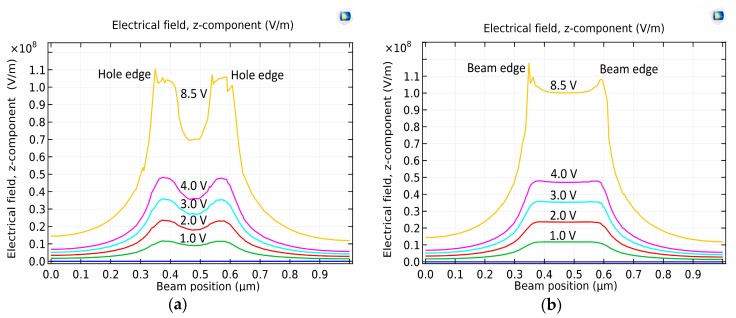
*z*-displacement of the electrical field at dissimilar applied voltages between: (**a**) gold bottom electrode and perforated graphene beam; (**b**) gold bottom electrode and intact graphene beam.

Besides, Figure 15 depicted the 1D electrical strength in the *z*-direction at the different applied voltages. The electrical field strength was extremely concentrated at the graphene beam edges and hole edges. These outputs reveal that the downward component of the electrostatic force stand-in on the hole edges and graphene beam edges, leading to the higher von Mises stress at the hole edge compared to the von Mises stress at the beam center.

## 4. Conclusions

In this article, we have studied the characteristics of electromechanical switching and mechanical stability of an intact and perforated graphene-based NEM switch by FEM simulations. In order to estimate their effect towards actuation pull-in voltage and von Mises stress, we had scaled the device dimensions namely *L*, *t*, *g*, *HL*, *HW*, *DL*, and *CN*. These analyses suggested an actuation pull-in voltage of 1.5 V can be achieved by the following dimension: graphene beam width of 0.3 µm with a length of 1.5 µm, a thickness of 3.0 nm, air gap thickness of 70 nm, hole length of 250 nm, hole width of 25 nm, distance of 100 nm between two holes, and 12 numbers of hole column of the graphene-based NEM switch. This simulated actuation pull-in voltage value of 1.5 V was comparable with the conventional semiconductor switch in the CMOS circuit that typically operated at 2 V. More efforts are still needed to further reduce the actuation voltage and enhance the mechanical stability of the graphene-based NEM switch. Parameters like air damping effect, Casimir effect, and van der Waals force are to be considered for the future NEM switch analysis.

## Figures and Tables

**Table 1:** Dimensions of the intact graphene beam NEM switch.

NEM Switch	Length, *L* (µm)	Thickness, *t* (nm)	Width, *W* (µm)	Air Gap Thickness, *g* (nm)	Pull-In Voltage (V) [20]	Pull-In Voltage (V)
i	1.5	9.0	0.3	75	8.5	8.4
ii	1.0			50	15.0	17.4
iii	0.8			45	22.0	21.8

**Table 2:** Dimensions of intact graphene-based NEM switch with variation of beam length (*L*), thickness (*t*), and air gap thickness (*g*).

NEM Switch	Remark	Length, *L* (µm)	Thickness, *t* (nm)	Air Gap Thickness, *g* (nm)	Width, *W_b_* (µm)	Pull-In Voltage (V)
i	Length, *L* variation	0.8	3	70	0.3	7.7
ii		1.0				5.0
iii		1.5				2.2
iv	Thickness, *t* variation	1.5	5	70	0.3	4.7
v			7			7.8
vi			9			11.5
vii	Air gap, *g* variation	1.5	3	100	0.3	3.4
viii				130		4.7

**Table 3:** Dimensions of the perforated graphene-based NEM switch with variation of hole length (*HL*), hole width (*HW*), distance between two holes (*DL*), and number of hole column (*CN*).

NEM Switch	Remark	Hole Length, *HL* (nm)	Hole Width, *HW* (nm)	Distance between Each Hole, *DL* (nm)	Number of Hole Column, *CN*	Pull-In Voltage (V)	Pull-In Voltage (V) Analytical
A	Hole length, *HL* variation	50	25	100	12	2.2	1.46
B		100				2.1	1.44
C		150				1.9	1.43
D		200				1.7	1.41
E		250				1.5	1.39
F	Hole width, *HW* variation	250	25	100	6	2.0	1.44
G			50			2.0	1.39
H			75			2.1	1.36
I			100			2.0	1.32
J	Distance between each hole, *DL* variation	250	25	25	12	2.0	N/A
K				50		1.9	
L				75		1.8	
M	Number of hole column, *CN* variation	250	25	100	8	1.9	1.42
N					10	1.8	1.41
